# Reprogramming barriers in bovine cells nuclear transfer revealed by single‐cell RNA‐seq analysis

**DOI:** 10.1111/jcmm.17505

**Published:** 2022-08-15

**Authors:** Lixia Zhao, Chunshen Long, Gaoping Zhao, Jie Su, Jie Ren, Wei Sun, Zixin Wang, Jia Zhang, Moning Liu, Chunxia Hao, Hanshuang Li, Guifang Cao, Siqin Bao, Yongchun Zuo, Xihe Li

**Affiliations:** ^1^ The State Key Laboratory of Reproductive Regulation and Breeding of Grassland Livestock, College of Life Sciences Inner Mongolia University Hohhot China; ^2^ Research Center for Animal Genetic Resources of Mongolia Plateau, College of Life Sciences Inner Mongolia University Hohhot China; ^3^ Inner Mongolia Saikexing Institute of Breeding and Reproductive Biotechnology in Domestic Animal Hohhot China; ^4^ College of Veterinary Medicine, Key Laboratory of Basic Veterinary Medicine Inner Mongolia Agricultural University Hohhot China; ^5^ Beijing Advanced Innovation Center for Genomics, College of Life Sciences Peking University Beijing China

**Keywords:** biPSCNT, 3F biPSCs, bovine, IVF, SCNT, single‐cell RNA‐seq

## Abstract

Many progresses have recently been achieved in animal somatic cell nuclear transfer (SCNT). However, embryos derived from SCNT rarely result in live births. Single‐cell RNA sequencing (scRNA‐seq) can be used to investigate the development details of SCNT embryos. Here, bovine fibroblasts and three factors bovine iPSCs (3F biPSCs) were used as donors for bovine nuclear transfer, and the single blastomere transcriptome was analysed by scRNA‐seq. Compared to in vitro fertilization (IVF) embryos, SCNT embryos exhibited many defects. Abnormally expressed genes were found at each stage of embryos, which enriched in metabolism, and epigenetic modification. The DEGs of the adjacent stage in SCNT embryos did not follow the temporal expression pattern similar to that of IVF embryos. Particularly, SCNT 8‐cell stage embryos showed failures in some gene activation, including *ZSCAN4*, and defects in protein association networks which cored as POLR2K, GRO1, and ANKRD1. Some important signalling pathways also showed incomplete activation at SCNT zygote to morula stage. Interestingly, 3F biPSCNT embryos exhibited more dysregulated genes than SCNT embryos at zygote and 2‐cell stage, including genes in KDM family. Pseudotime analysis of 3F biPSCNT embryos showed the different developmental fate from SCNT and IVF embryos. These findings suggested partial reprogrammed 3F biPS cells as donors for bovine nuclear transfer hindered the reprogramming of nuclear transfer embryos. Our studies revealed the abnormal gene expression and pathway activation of SCNT embryos, which could increase our understanding of the development of SCNT embryos and give hints to improve the efficiency of nuclear transfer.

## 1 INTRODUCTION

Somatic cell nuclear transfer (SCNT) can be used to reprogram a terminal differentiated nucleus to its totipotent state, and it has great potential for use in animal breeding, regenerative medicine and endangered species protection.^1^, ^2^, ^3^ Since the first cloned mammal ‘Dolly’,^2^ more than 20 mammalian species have been successfully cloned through SCNT technology.^4^, ^5^ Despite its success, several technical obstacles limit the application of SCNT technology, such as the low cloning efficiency, abnormalities observed in the placenta of the cloned embryos,^6^ and some abnormalities even observed in cloned animals after their birth.^7^, ^8^ These observations suggest the existence of barriers that prevent normal development of cloned animals. Therefore, elucidating the reprogramming barriers and finding effective methods to improve the efficiency of SCNT have become urgent problems to be solved.

In SCNT preimplantation embryos, abnormal gene expression patterns have been observed at the 2‐cell stage of mouse embryo, which corresponds to the major wave of zygotic genome activation (ZGA) in normal embryos.^9^ In mice, ~1000 genomic regions or genes failed to activate at ZGA in SCNT embryos. These reprogramming‐resistant regions or genes are enriched for the transcription repressive marker H3K9me3, which appears to be a general barrier in mammalian SCNT reprogramming.^10^ The H3K9me3 demethylase KDM4 mRNA injection can increase the cloning efficiency in many species,^4^, ^11^, ^12^ and abnormal genes can be partially reactivated by the Kdm4d mRNA injection. Moreover, transcriptional memory has been shown to exist in somatic cells and induced pluripotent stem cells (iPSCs).^13^, ^14^, ^15^

To date, many types of donor cells were used for the production of nuclear transfer animals. In addition to differentiated cells,^3^, ^16^ undifferentiated embryonic cells and embryonic stem cells nuclear transfer achieved success in bovine and other animals,^17^, ^18^ and iPSC as a donor also succeed generated cloned mice.^14^, ^19^ It seems that mammalian SCNT efficiency is inversely correlated with the differentiation status of donor cells. What about the bovine‐induced pluripotent stem cell (biPSCs)? In this study, 3F bovine iPSCs nuclear transfer (3F biPSCNT) was performed and 3F biPSCNT zygote and 2‐cell stage embryos were examined for their potential.

Recently, single‐cell RNA‐Seq (scRNA‐Seq) techniques have been used to analyse the transcriptome of many species' embryos at single‐cell resolution.^20^, ^21^ Here, using the scRNA‐Seq technique, we reported abnormal gene expression of bovine early cloned embryos, especially at 8‐cell stage of SCNT, embryos showed failures in the activation of a series of genes, including *ZSCAN4*, and defects in functional protein association networks. Moreover, we found 3F biPSCNT embryos had more dysregulated genes and has a different developmental fate from SCNT and IVF embryos, suggesting partial reprogrammed bovine iPS cells as donors for bovine nuclear transfer hindered the reprogramming of nuclear transfer embryos.

## 2 MATERIALS AND METHODS

### 2.1 Derivation of 3F biPSCs

China Qinchuan bovine fetal (day 45) fibroblasts (BFFs) were planted on gelatinized T75 culture flask and cultured in M10 medium. M10 medium formulation was as follows: knockout DMEM (Gibco, 10829‐018), 10% FBS (Gibco), 1 × Penicillin–Streptomycin (Gibco) and 1 × MEM Non‐Essential Amino Acids (Gibco). BFFs were dissociated with TrypLE™ Select (Gibco, 12563‐029) and harvested for electroporation at 80% confluence (~1.0 × 10^6^ cells per experiment). The transfections were performed using an Amaxa Nucleofector machine (Lonza) according to the manufacturer's protocol (Basic Nucleofector® Kit for Primary Mammalian Fibroblasts, VPI‐1001, program U‐23), with 0.5 μg PB–TRE‐b*cMYC* (bovine *cMYC*), 1.0 μg PB–TRE–hRL (human *RARG* and *LRH1*), 1.0 μg PB–EF1a–transposase and 1.0 μg PB–EF1a–rTTA.^22^ After transfection, 0.5 million BFFs were seeded on mitomycin‐inactivated BFFs feeders in M15 supplemented with LIF (10 ng/ml, Millipore, LIF1001), Vitamin C (Sigma, 49752), 10 ng/ml bFGF (R&D, 233‐FB‐025) and Dox (1.0 μg/ml, Clontech, 631311) in 10‐cm dishes. M15: knockout DMEM (Gibco, 10829‐018), 15% FBS (BI, 04‐002‐1A), 1 × Penicillin–Streptomycin (Gibco, 11140‐050), 1xGlutaMAX (Gibco, 35050‐061), 1 × MEM Non‐Essential Amino Acids (Gibco) and 0.1 mM 2‐mercaptoethanol (Sigma, M6250). The culture media was changed every other day, and the colonies were picked in M15 supplemented with Dox at day 15–20 and maintained in the same medium as described in our previous study.^23^

### 2.2 Character analysis of 3F biPSCs

Karyotyping and alkaline phosphatase activity analysis of bovine 3F biPSCs were according to our previous study.^24^ Quantitative Real‐Time PCR analysis of pluripotent gene expression in 3F biPSCs was as follows. Total RNA was isolated using a RNeasy Mini Kit (Qiagen, 74104) for cultured cells, and complementary DNA (cDNA) was prepared using a GoScript™ Reverse Transcription System (Promega，A5001). All RT‐qPCR reactions were performed on Veriti 96 cell Thermal Cycler (Applied Biosystems). RT‐qPCR primers were used as previously described.^23^ Gene expression was determined relative to GAPDH using the ΔΔCt method. Data are shown as the mean and SD.

### 2.3 In vitro fertilization

After 22–24 h of incubation, Groups of 50–60 COCs were transferred to four well plates containing 250 μl of BO‐IVF medium (ivfbiosience). Holstein semen was thawed in 37°C water bath for 20 s, following washed twice in 2 ml of BO‐Semen Prep medium (ivfbiosience) by centrifugation at 328 *g* for 5 min. The supernatant was discarded, and the sperm pellet containing viable spermatozoa was resuspended and diluted in the appropriate volume of BO‐IVF medium to achieve a concentration of 4 × 10^6^ sperm/ml. The sperm suspension (250 μl) was added to each fertilization well to obtain a final concentration of 2 × 10^6^ sperm/ml. Oocytes and sperm were coincubated for 18 h at 38.5°C under a humidified atmosphere of 5% CO_2_ in air. After fertilization of oocytes, cumulus cells were removed and the denuded oocytes were transferred in groups of 35–40 to four well plates containing 500 μl of SOF medium, under an atmosphere of 5% CO_2_, 5% O_2_, 90% N_2_ for further development.

### 2.4 Production of nuclear transfer embryos reconstructed with BFFs and 3F biPSCs

The BFFs within passages P3‐P6 and 3F biPSCs P15‐25 were dispersed to a single cell suspension by TrypLE select (Invitrogen) and recovered in M10 and M15+DOX, respectively. They were used as donor cells for nuclear transfer. The NT protocol was performed as previously described.^23^ Briefly, matured oocytes were denuded of cumulus cells, and oocytes with the first polar body were transferred into TCM199‐Hepes medium containing 7.5 μg/ml cytochalasin B. Enucleation was performed with a 20‐mm (internal diameter) glass pipette by aspirating the first polar body and approximately 5% of the adjacent surrounding cytoplasm. Single cells were individually transferred to the perivitelline space of the recipient cytoplasts. Cell fusion was performed using two direct current pulses of 1.0 kV/cm for 10 μs by an ECM 830 Electroporation System (BTX) in 0.30 M mannitol, 0.05 mM CaCl_2_, 0.1 mM MgCl_2_, and 0.05% BSA. Successfully reconstructed embryos were kept in modified synthetic oviductal fluid (mSOF) (containing 5 mg/ml cytochalasin B) for 2 h until activation. All fused embryos were further activated in 5‐mM ionomycin for 5 min, followed by exposure to 2 mM 6‐dimethylaminopurine in SOF for 4 h. After the activation, NT embryos were washed and transferred into 500 μl of SOF media covered with mineral oil in a 4‐well plate, under an atmosphere of 5% CO_2_, 5% O_2_, 90% N_2_ for further development.

### 2.5 Bovine embryo dissection and individual blastomeres, single‐cell isolation

The protocol was performed as previously described.^21^ Firstly, the zona pellucida of bovine embryos was removed using Tyrode's solution, Acid (Sigma). The denuded embryos were then treated with 0.02% EDTA for 5 min and placed into Accutase medium (Chemicon, SCR005 for morulae or blastocysts) or 0.005% trypsin (GIBCO, for zygote, 2‐cell or 8‐cell stage embryos) for 30–60 min. Single blastomeres were isolated by gentle, repeated pipetting. The morulae and blastocysts were placed in droplets for separate single‐cell treatment too. The single cells were picked after being incubated in Accutase for 30 min. When all of the blastomeres were separated, they were removed from the manipulation drops, washed 3–5 times in prewarmed PBS with 1% HSA medium, and placed into lysis buffer immediately for the preparation of the single‐cell cDNA library. BFFs and 3F biPSCs were dispersed to a single cell suspension by TrypLE select (Invitrogen), then single cells were put into the lysis buffer using a mouth pipette.

### 2.6 Single‐cell RNA‐seq library preparation and sequencing

Single‐cell RNA‐seq libraries were prepared by using a modified protocol based on the STRT‐seq protocol. Briefly, after tissue digestion, single cells were picked into 2 μl cell lysis buffer with a mouth pipette under a microscope. Reverse transcription was performed with oligo dT primers composed of 8 nt cell‐specific barcodes, 8 nt unique molecular identifiers (UMI) and 25 nt oligo dT. The second‐strand cDNA was synthesized followed by 19 cycles of PCR amplification with the 3’P2 primer and the IS primer. Then, 96 different barcoded single‐cell PCR pre‐amplified products were pooled together and purified by AMPure XP beads (Beckman). Forty nanograms of DNA was then used to process four cycles PCR with IS primer and biotin‐modified index primer. Index‐induced cDNA was sheared to ~300 bp fragments by Covaris S2. Fragmented DNA was then enriched by incubating with streptavidin C1 beads (Thermo Fisher) for 1 h. Finally, the libraries were constructed using a KAPA Hyper Pre Kit (KAPA Biosystems). All single‐cell RNA‐seq data were generated on an Illumina HiSeq4000 platform for 150‐bp paired‐end reads.

### 2.7 Processing of single‐cell RNA‐seq data

We separated raw reads based on the barcode information of the first 8 bp in reads 2 from the paired‐end reads. Then, the TSO sequence and polyA tail sequence in reads 1 were removed using a customized script and Trimmomatic,^25^ respectively. Subsequently, filter out low‐quality bases (N > 10%) or adapter contaminants (length < 37 bp) in read 1. The stripped read 1 sequence was then aligned to the bosTau9 cow reference genome (University of California, Santa Cruz, UCSC) using Hisat2.^26^ The output .sam files were converted to .bam files and sorted by samtools.^27^ Then, we used htseq‐count from the HTSeq package^28^ to count uniquely mapped reads, which were then grouped on the basis of the cell‐specific barcodes.

### 2.8 Uniform Manifold Approximation and Projection (UMAP) and clustering based on the expression matrix

The UMAP dimensionality reduction analysis and cell clusters were executed by monocle3^29^ R package. The monocle3 function reduce_dimension (reduction_method=“UMAP”) was used to execute unsupervised clustering analysis and the cluster_cells function was used to cluster cells. Then, top_markers function was used to identify unique cluster‐specific marker genes and the top 1 specific marker was selected by “specificity” parameter.

### 2.9 Identification of DEGs, KEGG pathways and GO terms

Seurat function find_all_markers (thresh. test=1, tes‐t.use=“roc”) was used to identify unique cluster‐specific marker genes. For two given clusters, DEGs were identified by the find.markers function with the following parameters: thresh.use=1, test.use=“roc”. For a certain gene, the roc test generated a value ranging from 0 (for “random”) to 1 (for “perfect”), representing the “classification power”. Genes with a fold change >2 or <0.5 and a power >0.4 were identified as DEGs.

The KEGG pathways and GO terms were enriched by clusterProfiler^30^ R packages. The functions “enrichKEGG” and “enrichGO” were used for KEGG pathways and GO terms enrichment analysis, and the pathways and terms with adjusted *p* value <0.05 were regarded as significantly enriched pathways and terms.

### 2.10 Functional protein association networks construction

The DEGs of I‐cluster 4 were imported into string (functional protein association networks, https://string‐db.org/). Then, the DEGs with the greatest number of nodes were defined as core factors.

### 2.11 Statistical analysis and data visualization

The Pearson correlation coefficient was calculated using the ‘cor’ function with default parameters to estimate the correlation between genes.^31^ The developmental data are represented as the average plus standard deviation of biological replicates (Mean + SD). Student's t test was performed using the ‘*t*.test’ function with default parameters.^32^, ^33^

In this study, data visualization was mainly achieved with R (version 3.6.3), including the R/Bioconductor (http://www.bioconductor.org) software packages. The heatmap and Venn plot were produced using R packages Pheatmap and VennDiagram, respectively. The density graph, boxplot, bubble chart, and so on were generated with the ggplot2 (http://ggplot2.org/).

## 3 RESULTS

### 3.1 Pluripotency of 3F biPSCs

**FIGURE 1:**
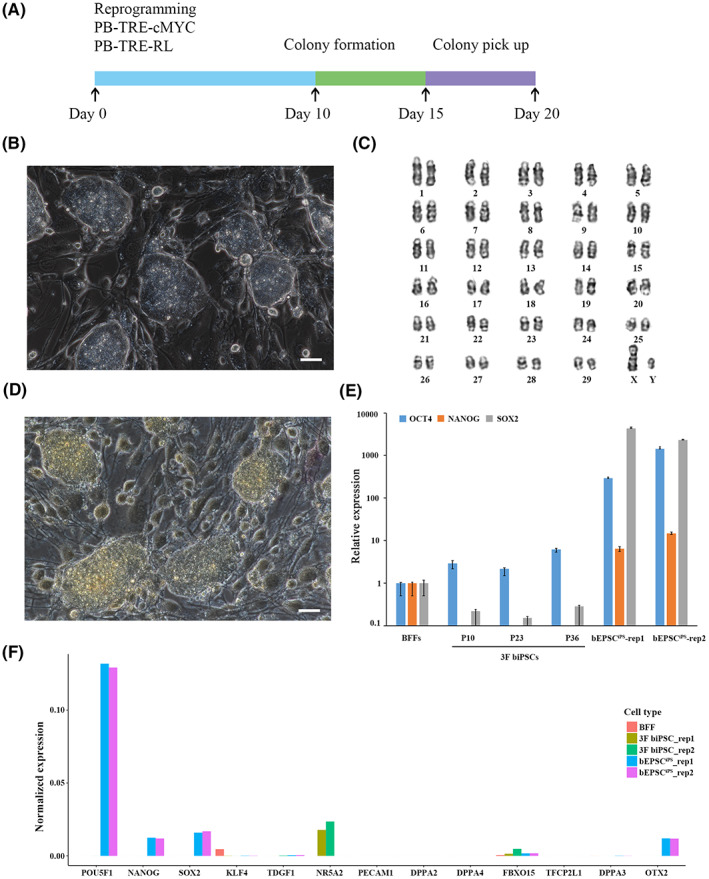
Derivation and character of 3F biPSCs. (A) Schematic illustration of reprogramming BFFs to 3F biPSCs. (B) The Domed mophorlogy of 3F biPSCs colony. (C) Karyotyping analysis of 3F biPSCs. (D) Negative AP staining of 3F biPSCs. (E) Relative expression of core pluripotency genes *OCT4*, *NANOG* and *SOX2* in different passages of 3F biPSCs on feeders. The relative expressions above were normalized to control BFF and housekeeping gene. Data represent the mean ± s.d.; *n* = 3 independent experiments. (F) Expression of pluripotency genes in 3F biPSCs, bovine EPSCs and BFFs of scRNA‐seq data. The gene expression values of different samples were normalized separately by read count

We expressed Dox‐inducible three exogenous reprogramming factors, b*cMYC* (bovine *cMYC*) and hRL (human *RARG* and *LRH1*) in bovine fetal fibroblasts (BFFs) of China Qinchuan bovine, delivered via *piggyBac* transposition and 3F biPSC colonies were picked on day 15 through 20 (Figure 1A). These 3F biPSCs could be maintained undifferentiated in Dox for at least 50 passages in a serum‐containing medium (M15+Dox) with domed morphology (Figure 1B). They were genetically stable and retained a normal karyotype (Figure 1C) (2*n* = 60, 36/50, 72%). These 3F biPSCs showed some defects in pluripotency, such as AP negative (Figure 1D) and with much lower expression of core endogenous pluripotent gene *OCT4*, *NANOG* and *SOX2*, compared to bovine expanded iPSCs (Figure 1E),^23^ suggesting nonactivation of endogenous pluripotent genes in 3F biPSCs. The same results were found in single‐cell RNA‐seq data (Figure 1F). Furthermore, these 3F biPSCs could not survive withdrawing Dox, suggesting their dependence on exogenous genes.

### 3.2 Transcriptional landscape across different bovine preimplantation embryos

**FIGURE 2:**
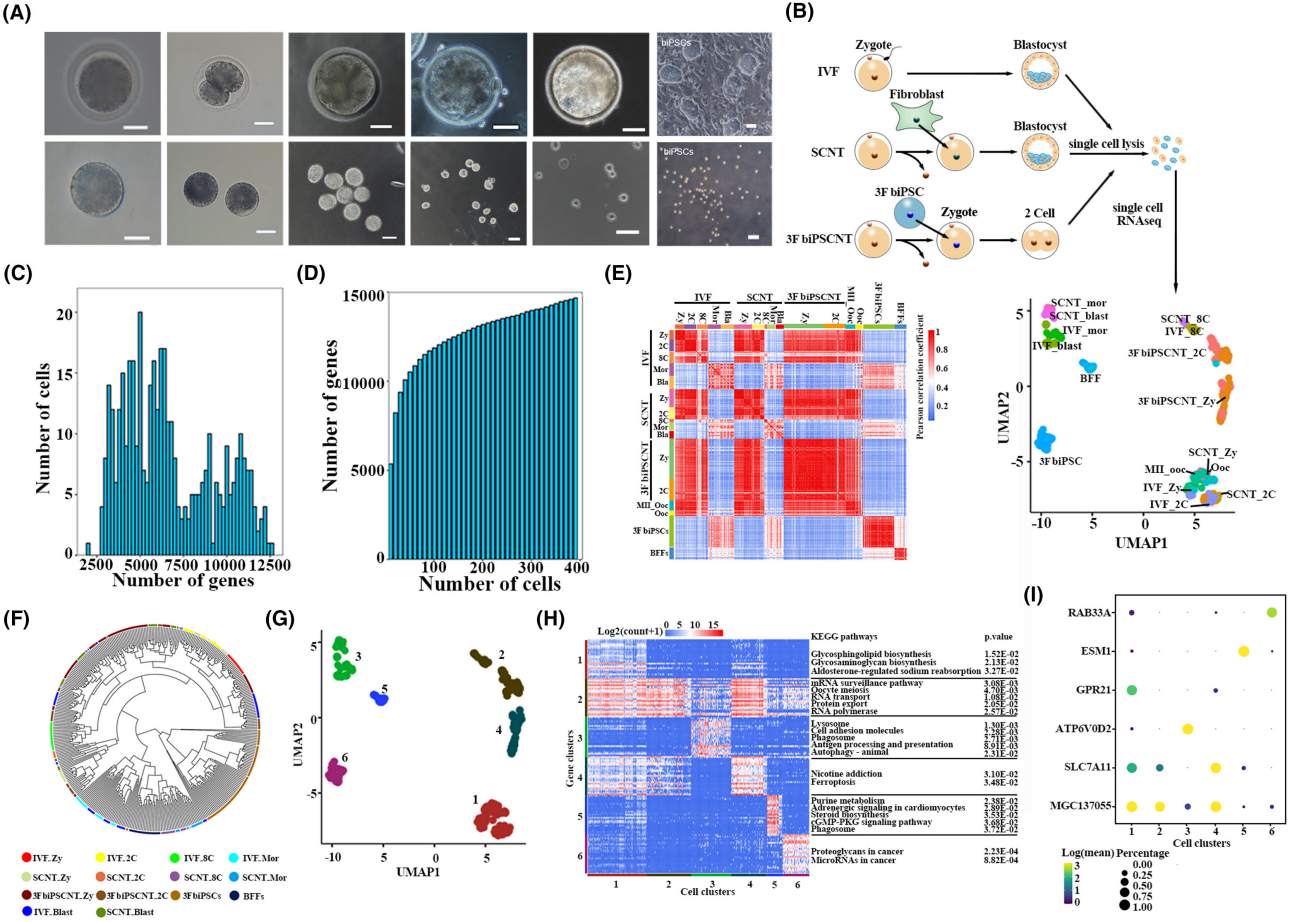
Single‐cell transcriptomic profiling of three types of bovine early embryos. (A) Microscopy imaging of bovine preimplantation IVF embryos at zygote, 2‐cell, 8‐cell, morula and blastocyst stages and their corresponding isolated single cells, 3F biPSC colonies, and their single cells. Scale bar, 50 μm. (B) Sampling of single cells from bovine embryos at zygote, 2‐cell, 8‐cell, morula and blastocyst development stages, as well as cDNA library preparation and single cell RNA sequencing. The dimension reduction analysis and cell clustering were based on the Uniform Manifold Approximation and Projection (UMAP) algorithm. (C) Distribution of the number of genes detected in 384 high‐quality single cells. (D) The cumulative number of genes detected in 384 high‐quality single cells. (E) Heatmaps showing Pearson correlation coefficients (PCC) between pairs of cells among each development stage and embryo type. (F) Hierarchical clustering among 384 single cells. (G) Based on the UMAP plot in Figure 2B, all single cells were divided into six clusters. (H) Heatmaps showing the expression patterns of 150 markers in six cell clusters, the enriched KEGG pathways listed right side. (I) Bubble chart exhibited the expression patterns of Top 1 marker in six cell clusters

Through our SCNT and IVF procedure, normal embryos were selected for analysis (Table S1). To compare the development between bovine SCNT and IVF embryos, we performed scRNA‐seq for 532 individual cells from 196 bovine oocytes, embryos and single cells, including unmatured oocytes, MII oocytes, IVF embryos (zygotes, 2‐cell stage embryos, 8‐cell stage embryos, morula and blastocysts), SCNT embryos (zygotes, 2‐cell stage embryos, 8‐cell stage embryos, morula and blastocysts), 3F biPSCNT embryos (zygotes and 2‐cell stage embryos), BFFs and 3F biPS single cells (+DOX) (Figure 2A, B). We generated 2.4 Tb sequencing data from 532 single cells. The average reads per cell were 2.92 million, and the reads length was 150 bp. After filtration, 384 high‐quality single cells were selected for subsequent analysis. The genes detected in these single cells were mostly around 5000, the average number was 6608, and the total number genes detected in these cells is 14,770 (Figure 2C,D). Then, we calculated the Pearson's correlation coefficient (PCC) between pairwise cells, and the result showed the cells at each stage of the embryos were highly correlated (average PCC > 0.65, Figure 2E). The PCC between the 8‐cell stage and morula of IVF and SCNT embryos were significantly reduced (average PCC < 0.5), corresponding to the obvious maternal‐zygotic transition (MZT) in gene expression pattern at this developmental stage.^34^

Then the dimension reduction and cell clustering based on the Uniform Manifold Approximation and Projection (UMAP) algorithm and cluster_cells function of Monocle3 were analysed, respectively. The results exhibited that the same cell types were mostly clustered together and the transcriptome of cells at each stage of SCNT embryos was close to that of IVF embryos (Figure 2B,F), then these cells were divided into six gene clusters (Figure 2G), and 150 markers were identified from all of the single cells by the “top markers” function of monocle3 (Figure 2H). The cluster 1 was specific expressed at zygote and 2‐cell stage of IVF and SCNT embryos, which was mainly enriched in the pathways of glycosphingolipid and glycosaminoglycan biosynthesis. The gene cluster 2 was specific expressed at 8‐cell stage of IVF and SCNT embryos, as well as 2‐cell stage of 3F biPSCNT embryos, which was mainly enriched in the transcription‐associated pathways, such as mRNA surveillance pathway, RNA transport, RNA polymerase. This was corresponding to the expression of a large number of zygote‐specific genes during ZGA. The gene cluster 3 was specific expressed at morula and blastocyst stage of IVF and SCNT embryos, which was mainly enriched in some transport and catabolism process, such as lysosome, phagosome, autophagy. The gene cluster 4 was specific expressed at zygote stage of 3F biPSCNT embryos only significantly enriched in some diseases‐associated pathways. Furthermore, we observed the most specific markers in each cell cluster (Figure 2I). *RAB33A*, a member of the RAS oncogene family, was specifically expressed in 3F biPSCs. *ESM1* as an endothelial cell‐specific marker was specifically expressed in BFFs. *ATP6V0D2*, which is involved in proton transmembrane transport, was specifically expressed at morula and blastocyst stages of IVF and SCNT embryos. *SLC7A11* is associated with transmembrane transporter activity and was specifically expressed in the zygote of 3F biPSCNT embryos.

### 3.3 Aberrant expression of genes in bovine SCNT embryos

**FIGURE 3:**
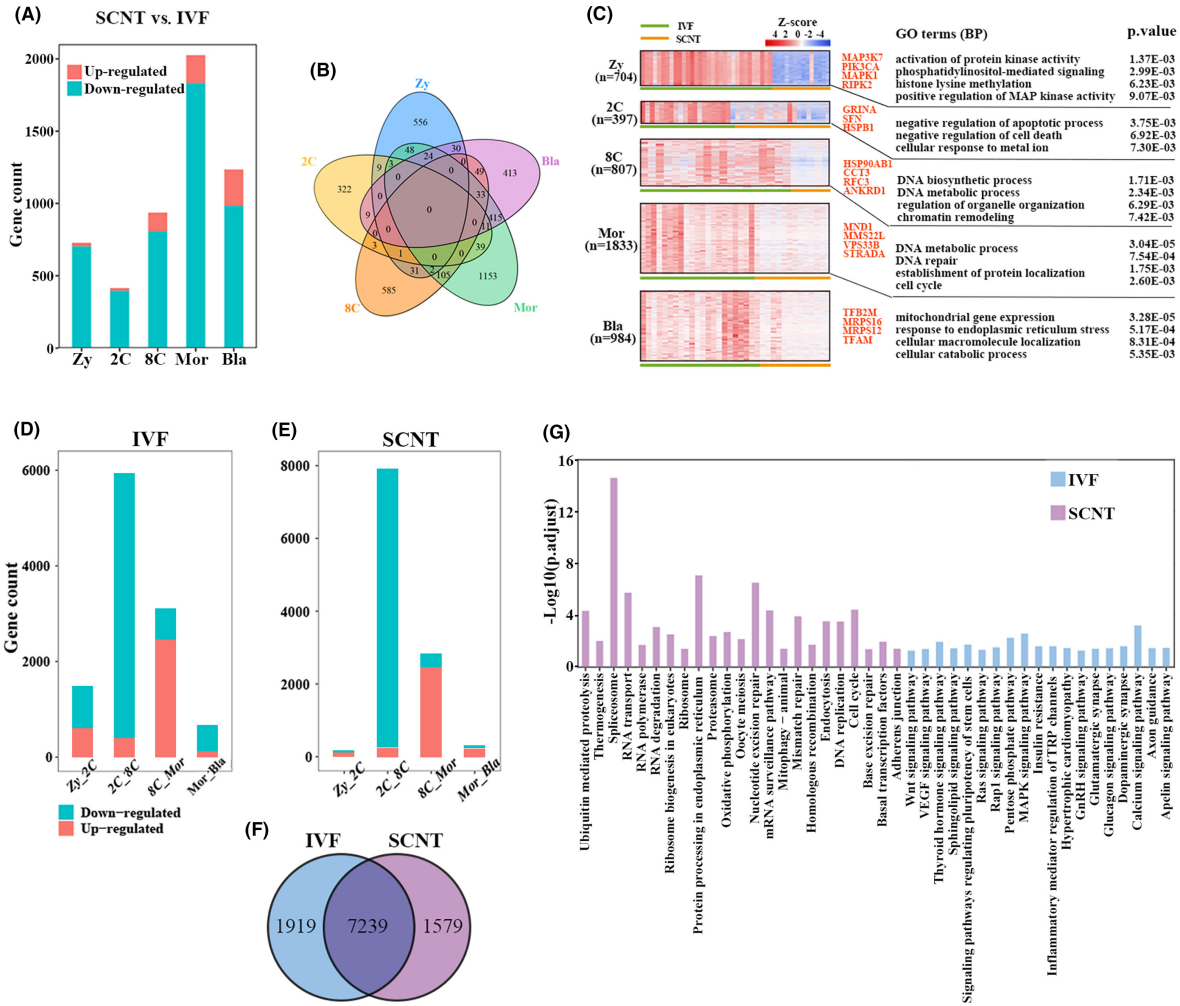
Different gene expression between SCNT embryos and IVF embryos. (A) Histogram represent the number of DEGs of each developmental stage in SCNT embryos compared to IVF embryos. (B) Overlaps among down‐regulated genes at each developmental stage (SCNT vs. IVF). (C) The expression patterns of down‐regulated genes (SCNT vs. IVF) in each developmental stage of IVF and SCNT embryos. The developmental stages and DEGs number were shown in the left. The enriched biological processes and representative genes were shown in the right. (D, E) Histogram represent the number of DEGs at the adjacent developmental stages of IVF and SCNT embryos, respectively. (F) Veen diagrams showing the overlapping of the overall DEGs among adjacent developmental stages between IVF embryos and SCNT embryos. (G) The enriched KEGG pathways of the specific DEGs in IVF embryos (*n* = 1919) and SCNT embryos (*n* = 1579)

The low efficiency of SCNT, the extraembryonic tissues abnormalities of the cloned embryos and some defects, including obesity, immunodeficiency, respiratory defects of cloned animals after birth were always found in SCNT animals.^7^, ^8^ In order to study the defects of bovine SCNT embryos, we performed differential expression analysis between SCNT embryos and IVF embryos (SCNT vs. IVF). Throughout the preimplantation stage, SCNT embryos had more down‐regulated genes than up‐regulated genes, compared to IVF embryos (Figure 3A). The down‐regulated genes exhibited stage specificity, and no consistently downregulated genes were identified (Figure 3B). Next, the expression patterns of down‐regulated genes and the enriched biological processes were showed in Figure 3C. The gene ontology (GO) terms enrichment analysis showed the aberrant biological processes of SCNT embryos at each development stage, such as activation of protein kinase activity, phosphatidylinositol‐mediated signalling, histone lysine methylation, positive regulation of MAP kinase activity at the zygote stage; negative regulation of apoptotic process, negative regulation of cell death at 2‐cell stage; DNA biosynthetic process, regulation of organelle organization, chromatin remodelling at the main ZGA (8‐cell stage) stage; DNA metabolic process, establishment of protein localization at the morula; mitochondrial gene expression, response to endoplasmic reticulum stress, cellular macromolecule localization at blastocyst stage.

Furthermore, in order to explore the dynamic changes of the transcriptome, we observed the change pattern of DEGs at the adjacent developmental stages of SCNT and IVF embryos. Although the number of DEGs at the adjacent embryo stages of SCNT and IVF embryos exhibited similar change pattern, and both reached the peak at the 2‐cell to 8‐cell stage (Figure 3D,E), the SCNT embryos down‐regulated DEGs at zygote to 2‐cell and morula to blastocyst stages were much less than that in IVF embryos. Interestingly, many genes between SCNT and IVF embryos have different expression patterns at each developmental stage (Figure 3A), but most DEGs from adjacent stages of SCNT and IVF embryos were shared (Figure 3F). These results suggested that the adjacent stage DEGs dynamically expressed in SCNT embryos were almost identical to those in IVF embryos, but lacked the same strict temporal expression pattern as in IVF embryos. In addition, SCNT embryos specific DEGs from adjacent stage were mainly enriched in processes related to RNA and DNA to maintain basic life activities, while IVF embryo‐specific DEGs from adjacent stage were mainly enriched in processes related to signalling pathway (Figure 3G), such as WNT signalling pathway, VEGF signalling pathway, Thyroid hormone signalling pathway, and signalling pathways regulating pluripotency of stem cells. The differential expression patterns of some important signalling transduction pathways were also observed, such as MAPK and Ras signalling pathways that can regulate cell proliferation and differentiation,^35^, ^36^ TGF‐β, WNT, and calcium signalling pathways which are related to morphogenesis or cell fate regulation.^37^, ^38^, ^39^, ^40^, ^41^ Partial genes of these five important signalling pathways had significant lower expression levels in SCNT embryos from the zygotic to morula stage. Until blastocyst stage, the gene expression of the key pathways reached the similar level to IVF embryos, implying that SCNT embryos may need to overcome the defects of these pathways to develop to the 8‐cell stage (Figure S1).

### 3.4 Bovine SCNT embryos exhibited functional protein association networks defect at the 8‐cell stage embryo

**FIGURE 4:**
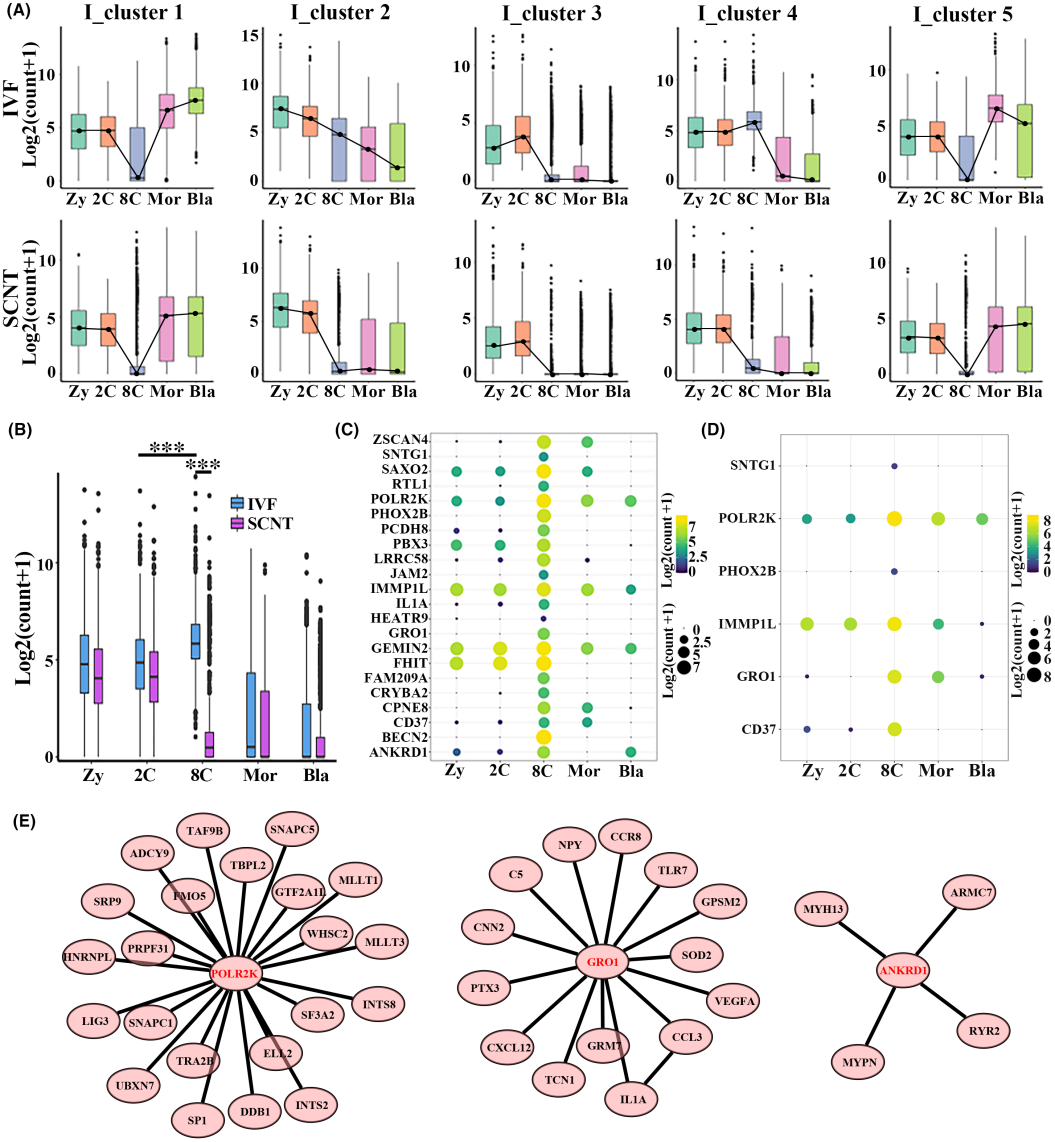
Abnormal gene expression of SCNT embryos at the 8‐cell stage. (A) Fuzzy c‐means (FCM) clustering analysis was used to divide the DEGs among adjacent developmental stages in IVF embryos and SCNT embryos into five clusters, respectively. (B) Boxplot showing the differential expression patterns of I_cluster 4 of IVF embryos between the two types of embryos. *p* < 0.001 taken as being statistically significant using Student's *t*‐test and denoted as ***. (C) The 22 most significant genes (power > 0.4, Avg_logFC > 1) in IVF embryos from I_cluster 4 selected by Seurat. (D) Only 6 out of the 22 most significance genes from I‐cluster 4 DEGs of IVF embryos were specifically expressed at the 8‐cell stage of SCNT embryos. (E) Three protein interaction networks based on I_cluster 4 were found by string (functional protein association networks) analysis

In order to further explore the gene expression asynchrony between bovine SCNT and IVF embryos, 9158 DEGs of IVF embryos as shown in Figure 3F were further analysed and assorted into five clusters based on fuzzy c‐means (FCM) clustering algorithm (Figure 4A). Gene cluster 1 of IVF embryos (I‐cluster 1) was specifically down‐regulated at 8‐cell. I‐cluster 2 was continuously down‐regulated. I‐cluster 3, I‐cluster 4, I‐cluster 5 were specifically up‐regulated at 2‐cell, 8‐cell and morula stage, respectively. We found I‐cluster 4 was up‐regulated at 8‐cell stage of IVF embryos, which down‐regulated at the corresponding 8‐cell stage of SCNT embryos (Figure 4B, *p* < 0.001). Then, the biological functions of these clusters were analysed (Table S2). The biological functions of I‐cluster 4 were enriched in cell division, regulation of cell cycle process, neural tube closure and tube closure, suggested these biological functions were abnormal in SCNT embryos, which maybe cause defects in further development of SCNT embryos.

Then, 22 markers of I‐cluster 4 with the highest specificity at the IVF 8‐cell stage embryo were found by Seurat^42^ (Figure 4C), including key gene for ZGA (*ZSCAN4*) and gene coding RNA polymerase subunit (*POLR2K*). But only six of these markers were expressed at the 8‐cell stage SCNT embryos (Figure 4D). Furthermore, the protein interaction network of the I‐cluster 4 was predicted by the string,^43^ and three regulatory networks cored as *POLR2K*, *GRO1* and *ANKRD1* were found (Figure 4E). POLR2K, GRO1 and ANKRD1 act as RNA Polymerase II transcription initiation and promoter clearance, G‐protein coupled receptor‐related protein binding and actin involved protein binding, respectively. These three regulatory networks were absent in SCNT 8‐cell stage embryo.

### 3.5 3F biPSNT embryo exhibited more defects at zygote and 2‐cell stage

**FIGURE 5:**
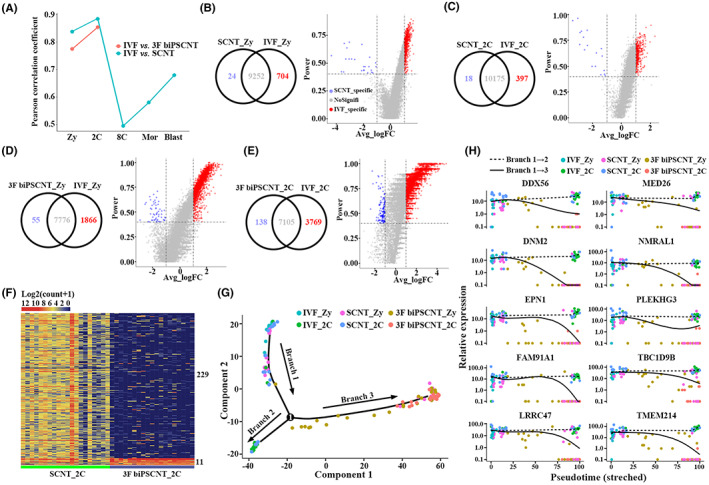
Compared with IVF embryos, 3F biPSCNT embryos showed greater differences than SCNT embryos. (A) A line graph of the Pearson correlation coefficients between cells of two types of nuclear transfer embryos and cells of IVF embryos at the corresponding developmental stage. **(**B–E) The venn diagrams and volcano diagrams showed the expression patterns of differentially expressed genes (DEGs) in SCNT embryos and 3F biPSCNT embryos compared to IVF embryos. (F) Expression patterns of differentially expressed genes between SCNT embryos and 3F biPSCNT embryos at the 2‐cell stage. (G) Pseudotime analysis of IVF, SCNT and 3F biPSCNT embryos from zygotes to the 2‐cell stage. The IVF and SCNT embryos appeared in branches 1 and 2, whereas 3F biPSCNT embryos appeared in branches 1 and 3. (H) The 10 genes that potentially regulated the development fate of nuclear transfer embryos were down‐regulated in 3F biPSCNT embryos from zygote to 2‐cell stage (branches 1–3) and have stable expression levels in IVF and SCNT embryos (branches 1–2)

To study the efficiency of nuclear transfer embryos using iPSC as donor cells, we performed evaluation for 3F biPSCNT embryos. The average paired Pearson correlation coefficients of single cells obtained from two types of nuclear transfer embryos at different developmental stages and IVF embryos at corresponding stages (Figure 5A). The results showed SCNT embryos were closer to IVF than 3F biPSCNT embryos at the zygotic and 2‐cell stage, suggesting 3F biPSCNT zygote and 2‐cell stage embryos had more heterogeneity. Next, Seurat was used to identify DEGs between NT and IVF embryos (Figure 5B–E). Compared to IVF embryos, 3F biPSCNT embryos had more down‐regulated genes than SCNT embryos at zygotes and 2‐cell stage (1866 and 3769, respectively), suggesting 3F biPSCNT embryos showed more defects in early embryonic development. Furthermore, the down‐regulated DEGs were much more than up‐regulated DEGs in SCNT and 3F biPSCNT embryos compared to IVF embryos (Table S3). The corresponding DEGs ratio was 704:24 and 397:18 at zygotes and 2‐cell stage of SCNT embryos, respectively. In 3F biPSCNT embryos, the ratio was 1866:55 and 3769:138, respectively. The more inhibited genes than activated genes suggested that SCNT embryos and 3F biPSCNT embryos were incomplete reprogramming, rather than erroneous reprogramming.

Two hundred forty DEGs (power > 0.8, |Avg_logFC| > 1) were found between SCNT embryos and 3F biPSCNT embryos at 2‐cell stage (Figure 5F). Among them, 229 SCNT up‐regulated DEGs mainly participate in biological processes such as collagen fibril organization, activation of GTPase activity, regulation of cytokinesis and protein heterotrimerization, and 11 3F biPSCNT down‐regulated DEGs involved in biological processes such as regulation of alternative mRNA splicing and mRNA processing. In order to explore the molecular reasons why 3F biPSCNT embryos showed more developmental defects, we further performed pseudotime analysis for IVF, SCNT and 3F biPSCNT embryos from the zygotes to the 2‐cell stage embryos based on monocle2.^44^ The results showed that the IVF/SCNT and 3F biPSCNT embryos bifurcated into two diverse branches from zygote to 2‐cell stage: the IVF and SCNT embryos appeared in branches 1 and 2, whereas 3F biPSCNT embryos appeared in branches 1 and 3 (Figure 5G). These results suggested 3F biPSCNT embryos have different developmental fate from IVF and SCNT embryos. Based on pseudotime analysis, we identified the genes that potentially regulated the developmental fate bifurcation of three types of embryos (Figure 5H). We found 10 genes (*DDX56*, *DNM2*, *EPN1*, *FAM91A1*, *LRRC47*, *MED26*, *NMRAL1*, *PLEKHG3*, *TBC1D9B* and *TMEM214*) were down‐regulated in 3F biPSCNT embryos from zygote to 2‐cell stage (branches 1–3) and had stable expression levels in IVF and SCNT embryos (branches 1–2). These 10 genes were all related to nucleic acid and protein binding, suggesting the abnormal process of transcription and translation may lead to developmental defect of 3F biPSCNT embryos.

**FIGURE 6:**
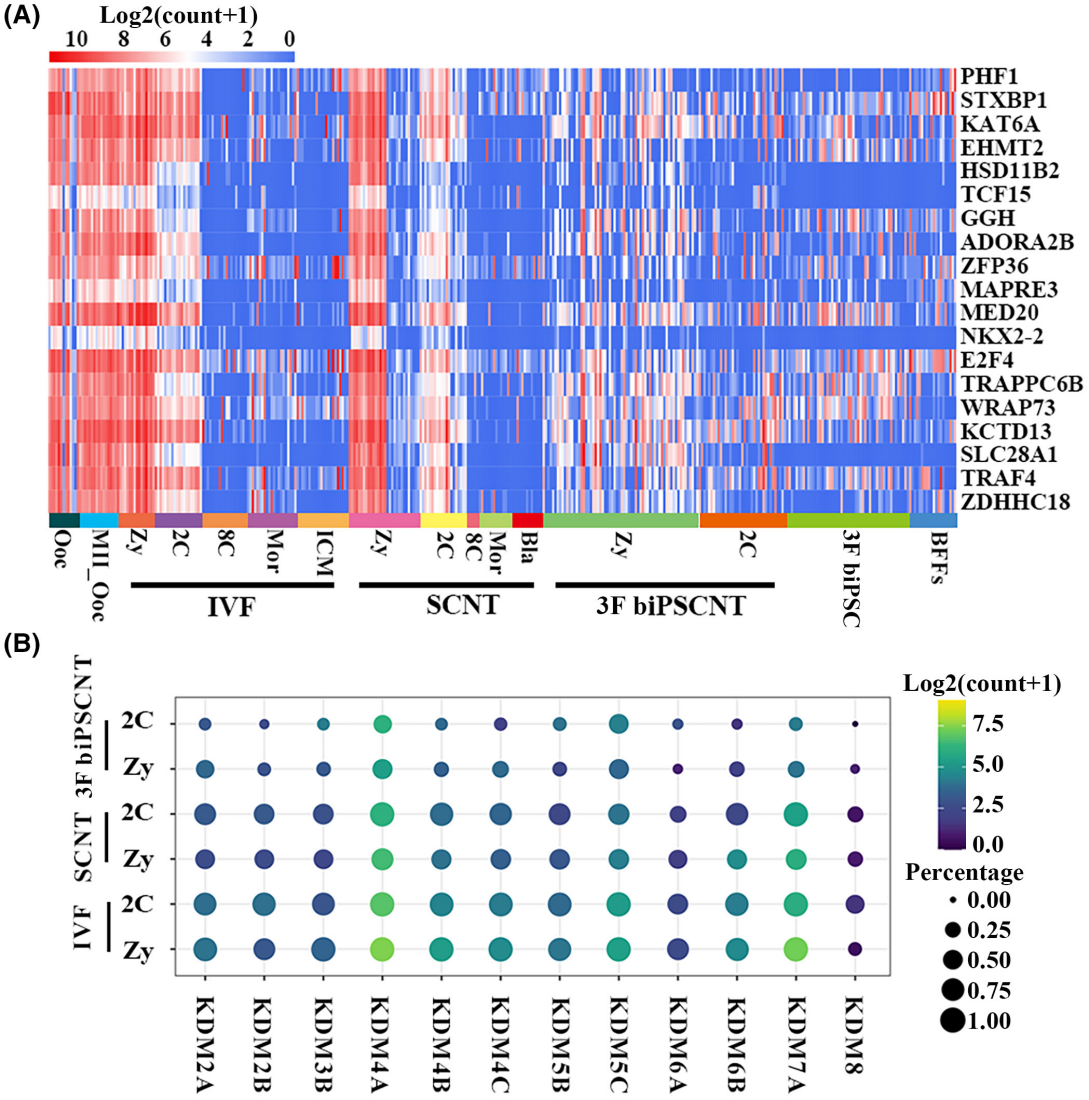
More defects of 3F biPSCNT zygote and 2‐cell stage embryos. (A) Heatmap showing the dynamic change of nineteen MII oocyte markers among the three types of embryos. (B) Expression patterns of the main lysine demethylases (KDMs) family at zygote and 2‐cell stage of IVF, SCNT and 3F biPSCNT embryos

Oocytes play an important role in SCNT‐mediated reprogramming, and oocyte extracts had been successfully used to mediate cell reprogramming.^45^ In order to explore the pioneering driving effects of key regulatory factors in oocytes, we identified 19 markers of oocytes in the MII period (power > 0.4, |Avg_logFC| > 1) by Seurat. These 19 oocytes markers had a high expression level in the zygote of IVF embryos, then begun to down‐regulated significantly at 2‐cell stage, then they were silenced after the 8‐cell stage embryos (Figure 6A). The similar expression pattern of these oocyte markers was observed in SCNT embryos, whereas in the 3F biPSCNT zygote at 8 and 16 h after nuclear transferring, these markers begun to down‐regulated.

Recently, many studies had shown that the ectopic expression of the main lysine demethylases (KDMs) family in mouse embryos can greatly improve the efficiency of SCNT.^9^, ^11^, ^46^ KDM5B mRNA injection significantly improved the development of bovine SCNT embryos.^47^ We next observed the expression patterns of KDMs family in bovine IVF, SCNT and 3F biPSCNT embryos (Figure 6B). The results showed that *KDM2A*, *KDM2B*, *KDM3B*, *KDM4A*, *KDM4B*, *KDM4C*, *KDM5B*, *KDM5C*, *KDM6A*, *KDM6B*, *KDM7A*, *KDM8* were differentially expressed in the three types embryos, suggesting histone reprogramming in zygotes and two cell embryos. Compared with SCNT and IVF embryos, the expression levels of KDMs in 3F biPSCNT embryos were significantly reduced, suggesting partial reprogramed iPSCs donors maybe increased the difficulty of reprogramming of NT embryos.

## 4 DISCUSSION

SCNT technology holds great potential for animal cloning, stem cell biology and therapeutic applications. In the past decade, many efforts have been made to improve cloning efficiency.^10^ The analysis of bulk samples has found large scale transcriptome differences between IVF and SCNT embryos, which were most evident at the 8‐cell and morula stages.^48^, ^49^ However, the transcriptome differences between IVF and nuclear transfer embryos at the single‐cell level and developmental defects of nuclear transfer embryos caused by such differences still remains largely unknown. On the other hand, the averaging of populations of cells tends to mask infrequent events or to overemphasize irrelevant biological processes not required for SCNT embryos development. Recently, single‐cell RNA sequencing (scRNA‐seq) technique is possible to analyse alterations in gene transcription within highly heterogeneous embryos at single‐cell level. Therefore, this technique could be particularly helpful for identifying molecular mechanisms involved in improving cloning efficiency of SCNT.

The single cells between IVF, SCNT, and 3F biPSCNT embryos at each stage were clustered together, suggesting the reliability of the single cell RNA‐Seq data. Although as shown in Figure 2A, SCNT embryos were similar to IVF embryos and most of the genes expressed were the same, we found abnormal expression patterns in bovine SCNT embryos. The gene ontology (GO) terms enrichment analysis showed the unique functional loss of SCNT embryos at each development stage, such as activation of protein kinase activity, phosphatidylinositol‐mediated signalling, histone lysine methylation, positive regulation of MAP kinase activity at the zygote stage; negative regulation of apoptotic process, negative regulation of cell death at 2‐cell stage; DNA biosynthetic process, regulation of organelle organization, chromatin remodelling at the major ZGA stage; DNA metabolic process, establishment of protein localization at the morula; mitochondrial gene expression, response to endoplasmic reticulum stress, cellular macromolecule localization at blastocyst stage. Li et al. showed that *Zscan4c*, which is critical for ZGA, is low expressed in mouse nuclear transfer arrest embryos.^50^ We observed *ZSCAN4C* down‐regulated in bovine SCNT embryos at major ZGA stage compared to IVF embryos.^51^ Especially, the missing functional protein association networks at the SCNT 8‐cell stage embryo, which networks were further analysed, and various binding function, including protein binding, DNA binding and metal ion binding were found, such as RNA polymerase II transcription factor binding function, suggesting the main ZGA was abnormal in SCNT embryos, as reported by other groups.^52^, ^53^

Successful generation of cloned mice using iPSNT had been reported,^19^, ^54^ in order to explore the potential of the 3F biPSCs as the donor for nuclear transferring, 3F biPSCNT were done, and the results were not optimistic. We found more defects in 3F biPSCNT zygote and 2‐cell embryo, and 3F biPSCNT embryos showed more dysregulated genes than SCNT embryos when compared to IVF embryos. Furthermore, the down‐regulated DEGs were much more than up‐regulated DEGs in SCNT and 3F biPSCNT embryos compared to IVF embryos. The corresponding DEGs ratio was 704:24 and 397:18 at zygotes and 2‐cell stage of SCNT embryos, respectively. In 3F biPSCNT embryos, the ratio was 1866:55 and 3769:138, respectively. The number of inhibited genes more than activated genes suggested that SCNT embryos and 3F biPSCNT embryos were incomplete reprogramming, rather than erroneous reprogramming. The pseudotime analysis of IVF, SCNT and 3F biPSCNT embryos from the zygotes to the 2‐cell stage embryos exhibited that SCNT embryos undergone a similar developmental process as IVF embryos, whereas different in 3F biPSCNT embryos. We identified 10 genes were down‐regulated in 3F biPSCNT embryos from zygote to 2‐cell stage and had stable expression levels in IVF and SCNT embryos. These genes mainly involved in the process of translation and transcription (*DDX56*, *LRRC47*, *MED26*),^55^ cell growth (*DNM2*),^55^ endocytosis (*EPN1*),^56^ apoptotic (*TMEM214*),^55^ and some genes whose functions have not been reported in nuclear transfer embryos (*FAM91A1*, *NMRAL1*, *PLEKHG3*, *TBC1D9B*), suggesting the defect of related biological processes in 3F biPSCNT embryos. We also found the expression pattern of oocyte makers were different in 3F biPSCNT embryos, whereas the similar expression pattern was found in SCNT and IVF embryos. For lysine demethylases (KDMs), their expression levels in 3F biPSCNT embryos were significantly reduced, suggesting partial reprogramed iPSCs donors maybe increased the difficulty of reprogramming of NT embryos. In the future, beyond that zygote and 2‐cell stage, other stage embryos including 8‐cell stage, morula and blastocyst stage of 3F biPSCNT embryos and other biPSCs induced by different factor combinations NT embryos should also be checked.

## AUTHOR CONTRIBUTIONS

**Lixia Zhao:** Data curation (equal); methodology (equal); writing – original draft (lead). **Chunshen Long:** Software (equal); visualization (lead). **Gaoping Zhao:** Investigation (equal); methodology (equal); resources (equal). **Jie Su:** Investigation (equal). **Jie Ren:** Data curation (equal). **Wei Sun:** Investigation (equal). **Zixin Wang:** Software (equal). **Jia Zhang:** Software (equal). **Moning Liu:** Resources (equal). **Chunxia Hao:** Formal analysis (equal). **Guifang Cao:** Validation (equal). **Siqin Bao:** Project administration (equal). **Yongchun Zuo:** Supervision (equal). **Xihe Li:** Project administration (equal); supervision (equal); writing – review and editing (equal). **Hanshuang Li:** Visualization (equal); writing – review and editing (equal).

## CONFLICT OF INTEREST

All authors have agreed to the publication of this paper and declare no potential conflicts of interest.

## Supporting information


Figure S1

Table S1

Table S2

Table S3

## Data Availability

The single‐cell RNA‐Seq raw data has been deposited to the Sequence Read Archive (SRA) database with accession code PRJNA727165.
