# Supplementary material for: Reprogramming barriers in bovine cells nuclear transfer revealed by single‐cell RNA‐seq analysis
Source: J Cell Mol Med. 2022 Aug 15;26(18):4792–804. doi: 10.1111/jcmm.17505 (PMC9465183; doi:10.1111/jcmm.17505)
Supplement: Supplementary file 1 — Figure S1 [file JCMM-26-4792-s003.docx]

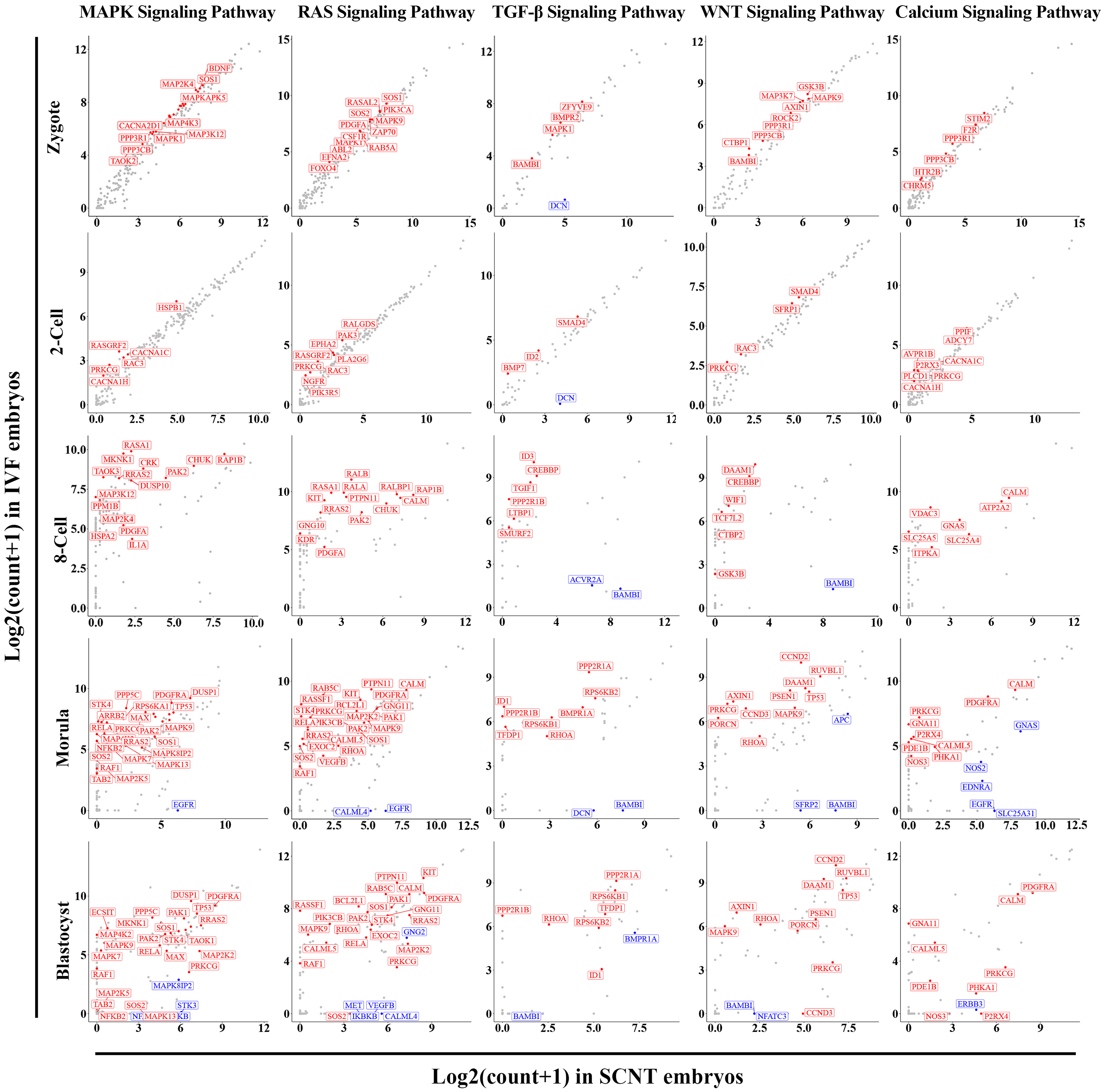


**FIGURE S1** Expression patterns of key signaling pathways in SCNT and IVF embryos at each development stage. The red dots represent the genes which were up regulated in IVF embryos. The gray dots represent the genes which were no significant differentially expressed. The blue dots represent the genes which were up regulated in SCNT embryos.
